# A “Salt and Pepper” Noise Reduction Scheme for Digital Images Based on Support Vector Machines Classification and Regression

**DOI:** 10.1155/2014/826405

**Published:** 2014-08-17

**Authors:** Hilario Gómez-Moreno, Pedro Gil-Jiménez, Sergio Lafuente-Arroyo, Roberto López-Sastre, Saturnino Maldonado-Bascón

**Affiliations:** Departamento de Teoría de la Señal y Comunicaciones, Universidad de Alcalá, Alcalá de Henares , 28805 Madrid, Spain

## Abstract

We present a new impulse noise removal technique based on Support Vector Machines (SVM). Both classification and regression were used to reduce the “salt and pepper” noise found in digital images. Classification enables identification of noisy pixels, while regression provides a means to determine reconstruction values. The training vectors necessary for the SVM were generated synthetically in order to maintain control over quality and complexity. A modified median filter based on a previous noise detection stage and a regression-based filter are presented and compared to other well-known state-of-the-art noise reduction algorithms. The results show that the filters proposed achieved good results, outperforming other state-of-the-art algorithms for low and medium noise ratios, and were comparable for very highly corrupted images.

## 1. Introduction

Nowadays the well-known machine learning tools (Support Vector Machines, Neural Networks, etc.) are used in high level digital image processing applications with high success. These high level applications include, for example, face recognition [1], traffic sign detection [2, 3], medical imaging [4], and more. However, the use of these tools in low level image processing is less extended. In this work we want to show how one of these tools, Support Vector Machines, can be used successfully in a low level image processing task as impulse noise removal.

Noise removal is one of the most important areas within digital image processing. One type of noise that can appear in images is impulse noise, which can be produced during image acquisition, storage, or transmission and can affect later stages of processing if not removed properly while preserving the details [5–7]. This problem arises in many fields, from medical imaging [8] to the analysis of satellite images [9].

Impulse noise appears when some of the pixels in the image are replaced by outliers while the rest remain unchanged. Outliers can have a fixed minimum or maximum gray-scale value or may vary within that range. The first type is known as “salt and pepper” and is the one analyzed in this paper.

Since reducing this noise presents a classical image processing problem, a great number of methods to reduce it have been proposed. In some methods every pixel in the image is restored regardless of whether it was originally noisy or not. The general scheme of this type of filtering is the use of a mask which is normally centered around the pixel of interest for computational reasons. The mask is used to sweep the image and perform some operations with the pixels inside it in order to obtain the reconstruction value.

This group includes the median filter, which is the classical solution for removing impulsive noise. This has been followed by a modification of the median filter, known as the center weighted median (CWM) filter [10, 11] which repeats the central pixel a number of times before ordering. To improve median performance, several authors (see [12, 13]) have proposed a filter which is closely related to the latter, called the adaptive median filter (AMF). This filter improves the performance of the CWM by using different mask sizes when the noise ratio is high.

An alternative to the filters using median may be presented in [14] known as minimum-maximum exclusive mean (MMEM) that is intended to be used in high noise ratios and is based on the mean of the neighbor values that are not considered as noisy in a 3 × 3 or 5 × 5 window.

The main advantage of these schemes is their simplicity, but the disadvantage is blurring, especially when the percentage of noise is high. This effect occurs because the filter changes both noisy and nonnoisy pixels. To prevent this problem, a previous noisy pixel detection or decision stage is required.

The scheme with a previous detection is commonly known as the switching scheme [5]. In this scheme only those pixels detected as noisy are reconstructed in any way, while the nondetected ones are maintained unchanged. Thus, the reconstruction effort is only applied to a reduced set of pixels and the process is quicker and more effective.

Most of the algorithms proposed in the literature are based on this scheme, since reconstruction is only applied to noisy pixels and the results are improved by reducing the blurring. The various methods proposed in this category vary the type of detector used and the means by which the noisy pixels are replaced.

Detection can be understood as a classification between noisy and nonnoisy pixels and has been implemented in several ways in state-of-the-art algorithms. Some of the proposed algorithms are based on median filtering [15, 16] like progressive switching median filter (PSMF) [17]. Rank ordered mean (ROM) filters are modifications of median filters used in [5, 18].

Recently some decision filtering methods are proposed like Decision Based Algorithm (DBA) [20] or modified decision based unsymmetric trimmed median filter (MDBUTMF) [24]. They are based on a decision scheme with no explicit classification. These approaches, that use local information, make that the restored image for high density noise contain several noisy spots. Other methods are based on similarity between pixels in a window to perform classification like [25]. An important group of detection-based schemes uses fuzzy logic [21, 22] or neural networks [26, 27].

This paper presents new impulse noise removal filters for application to “salt and pepper” noise. These filters use the switching scheme and the SVM, both for noise detection using classification and for reconstruction using regression.

Although the use of some training has been used in previous work [5], normally this training is based on real images or portions of them. The use of these real images can bias the results obtained and training cannot be controlled in every aspect. This is the reason why we propose the use of synthetic images for training. The synthetic images are potentially adaptable to the noise reduction problem.

The use of SVM is due to its good features in training and generalization. This is not the only classification tool available but the fact that the classification function obtained is unique for a given data train and that the machine complexity is obtained in the training process instead of given a priori size (like in neural nets) [28] makes the SVM a good choice for this type of problem. Another important feature that is desirable for this task is the generalization ability of the SVM when the training data are limited. In this way we can generate few training patterns, but expect a good degree of generalization with reduced training time.

Impulse noise detection using SVM is presented in Section 2, while in Section 3 a method to recover noisy pixels using SVM regression is presented and discussed.  Section 4 presents three new filters based on the classification and regression explained in the previous sections, and some experiments and results are then presented in Section 5. This section also describes how training affects detection and regression, enabling us to improve the results in our previous studies, and presents a comparison with some other state-of-the-art methods, confirming the quality of the proposed filters. Finally, some conclusions are discussed in Section 6.

## 2. Impulse Noise Detection Using SVM Classification

Following the switching scheme, we propose using a specifically designed classification algorithm to detect noisy pixels. SVM were chosen because of their capacity for generalization from a reduced set of training examples and better performance than other classification methods [29]. Preliminary versions of this idea were previously presented in [30], where we applied a modified median filter, which was then combined with regression in [31]. Due to the good results obtained in these previous studies, here we present a combined study of both noise detection and regression. Our proposal works by scanning the image using a window of a given size (usually 3 × 3) around the pixel to be classified as noisy or not. This 3 × 3 window is mapped left-to-right and top-to-bottom in a 1D vector of 9 components. Then, this vector is classified according the previously trained SVM classification function.

### 2.1. Synthetic Training Images for Noise Detection

In this study, the training vectors were generated from synthetic images (scanned as explained above), which have been designed to represent a wide range of gray values and edges of different levels. The optimization of the design of these training images is still an open question. The only requirements that must be met are the presence of a wide range of gray values and, at least, one edge. The objective was to generalize the training obtained to real images.

**Figure 1:**
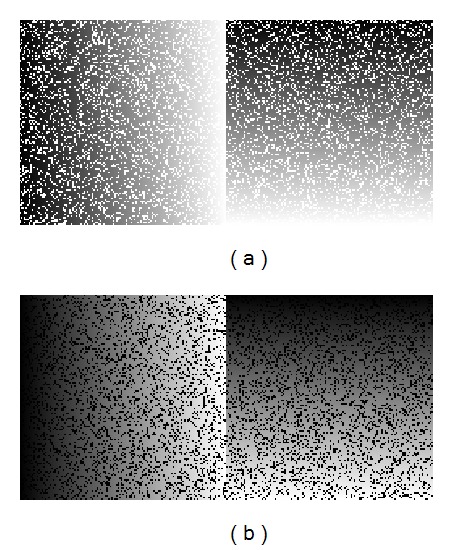
Example of training images for classification. The added impulse noise is 20%. (a) Training image to detect white pixels; (b) training image to detect black pixels.

Figure 1 gives an example of these synthetic training images. As shown, they can be subdivided into two similar subimages with an edge between them. Several edge configurations were tested and the best results have been obtained with the nonfix edge shown in Figure 1. There are two parameters to be configured in the training image generation process.

- *Image Height (H).* The image height (in pixels) modifies the different gray levels since these levels were obtained with a step between values equal to 256/*H*. Bigger images have a wider gray range which is more realistic.
- *Percentage of Added Noise (N).* We can add noise in different ratios (%) to best fit the real ratio in the image to be reconstructed.

**Figure 2:**
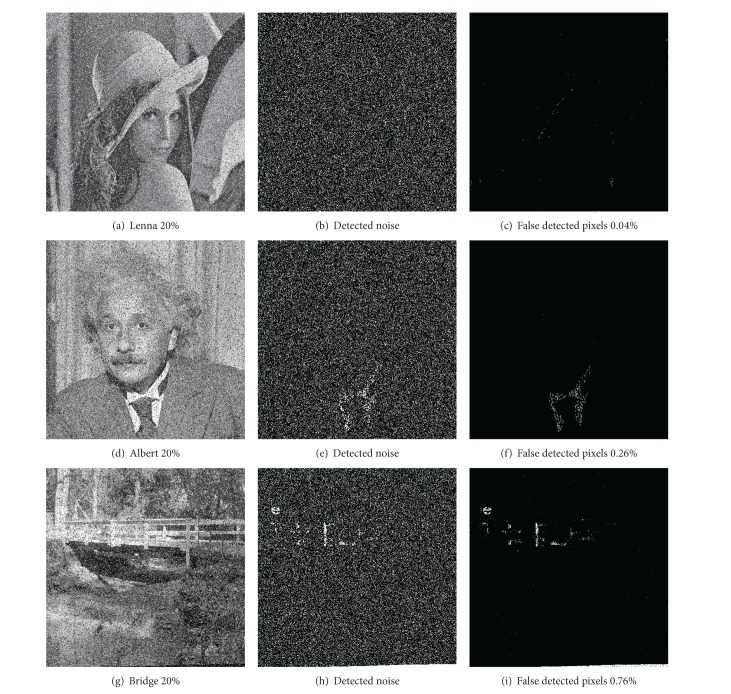
Examples of noise detection for images Lenna and Albert.

These parameters can be tuned to obtain the best classification results with the minimum number of support vectors.  Section 5.5 details the results obtained after an exhaustive tuning process, and Figure 2 shows examples of noise detection described in more detail in Section 5.4.

## 3. Impulse Noise Reconstruction Using SVM Regression

Once the noisy pixels have been detected, a reconstruction method can be implemented. One option would be to use a median filter or any of its modifications [30]. However, as presented in [31, 32], SVM regression is another option to recover image pixel values.

The regression scheme proposed uses only those pixel values around the pixel detected as noisy and excluding this one. The reason for using SVM regression is its good capacity for generalization from a reduced training set. The window size (neighborhood) was empirically fixed to 3 × 3, since this yielded the best results. Once again, the image is scanned using a window read top-to-bottom and left-to-right yielding an 8-components 1D vector.

In order to simplify the regression problem, only white pixel reconstruction is used since, in initial experiments, mixed white and black noise was difficult to recover, increasing the number of support vectors required and reducing the quality. The black pixels, once detected, are moved to white if regression is used.

### 3.1. Synthetic Training Images for Regression

**Figure 3:**
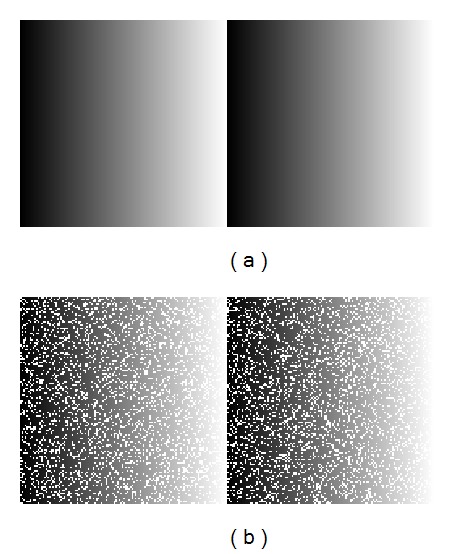
Example of a regression training image with a height of 128 and 20% added noise. (a) Nonnoisy image taken as model. (b) Noisy image with 20% white impulses.

The regression training is again based on synthetic images (similar to those used for classification and with the same *H* and *N* parameters). However, in this case two images are needed, one without noise which is used to obtain reconstruction values and one with noise which is used to obtain the training vectors. One example of these images is shown in Figure 3. These images contain gray values from 0 to 255 and a central edge and, as in classification training, the range of gray values depends on the image size. The main difference with classification training is that the original image is necessary and that the central edge was fixed white-to-black since the best results were obtained using this configuration. Once again, the optimization of these synthetic images is an open question.

Table 1 shows some lines in a file used for regression training in LIBSVM [33] format. Each line represents a training vector and its associated value. The first column is the original nonnoisy pixel, that is, the desired value for reconstruction. This value is obtained from the training images without added noise (see Figure 3(a)). The other columns are the vector components (numbered 1, 2, etc.) where the first one corresponds to the upper left pixel in a 3 × 3 window and so on. These vectors are obtained from the noisy image (see Figure 3(b)).

**Figure 4:**
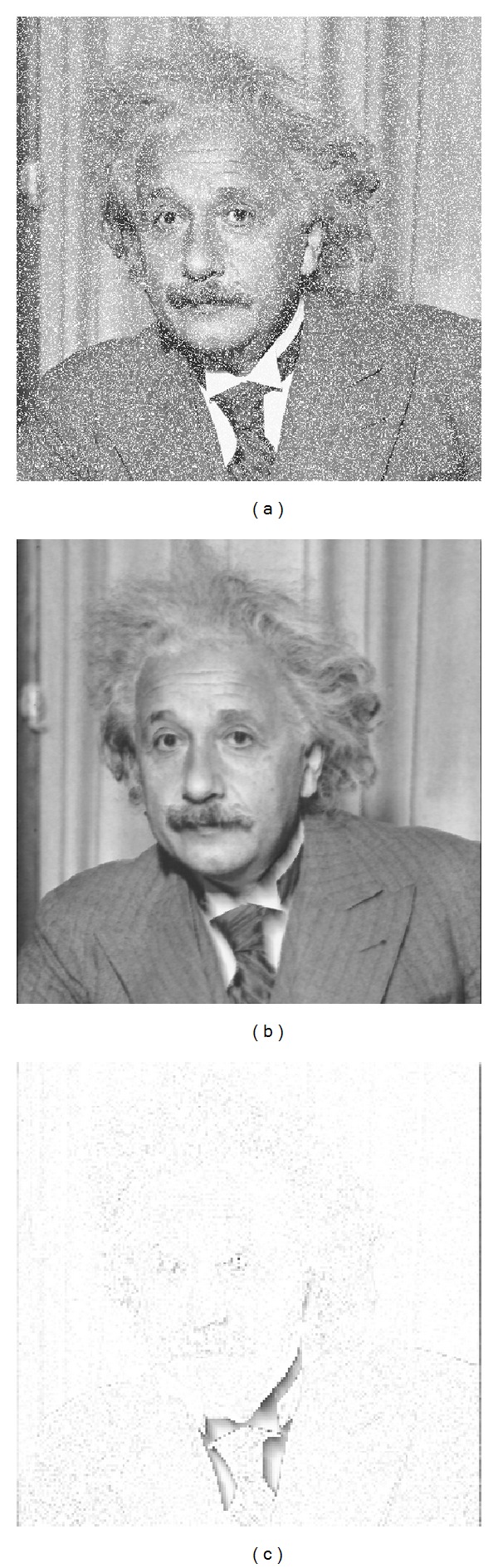
Regression example. (a) 20% noisy image; (b) reconstructed image; (c) difference with original (to improve visualisation, the darker values correspond to higher differences).

This is an automatic and configurable training (noise and size) but the results obtained are good with basic training parameters. For example, using a Gaussian kernel with *γ* = 3 · 10^−5^, *C* = 1000, and *ε* = 10^−3^ (see Section 5.7), we can obtain results like those shown in Figure 4 for Albert image. In these examples only regression was applied and we can see a certain degree of blurring [32]. This effect prevents the use of regression alone to reduce noise in images (especially for high noise ratios) although, as shown, the differences with original images are minimal. The differences appear in edges and in black or white homogeneous zones, since previously reconstructed values are used to obtain new ones. A detailed tuning process of the parameters is described in Section 5.7.

## 4. Proposed Filters Based on SVM Detection and Regression

Having described the detection and regression methods using SVM, we now propose some impulse noise reduction filters based on these methods. The filters proposed use SVM noise detection and the reconstruction method used establishes the differences between them.

In addition, there are two possible methods for implementing the proposed filters.

- *Recursive Implementation*. In this case, the pixels reconstructed previously are used to obtain new reconstruction values. In a 3 × 3 window this means that the first 4 values have been previously reconstructed while the last 4 have not. Thus, only one image sweep is required to eliminate noise.
- *Nonrecursive Implementation*. Only the gray values in the noisy image are used to obtain reconstruction values. In this case, it is possible to obtain isolated, unreconstructed noisy pixels. This effect is increased with high noise ratios. Thus, to obtain good results the noise reduction procedure must be applied several times in an iterative way. The iteration procedure is maintained until a 0.1% noise percentage is reached. This procedure is possible since our system gives the noise ratio actually present in the image.

### 4.1. Modified Median Filters

In these filters, the median is used to obtain the reconstruction values. The median is applied to the pixels around the detected noisy pixel or to a subset of them. Thus we have two possibilities.

1. The median is applied to the pixels detected as noisy and the central pixel is excluded since it is known to be noisy. This idea was presented previously in [5] and used with SVM in [30]. This method is called SVM-M1.
2. Since we can classify the pixels as either noisy or nonnoisy, when using the median, only the pixels detected as nonnoisy are included and, this way, isolated noisy pixels after reconstruction are reduced. This method is called SVM-M2.

The main difference between these is that when using SVM-M1, only one sweep is needed since detection and reconstruction are performed in the same step. However, with SVM-M2, one sweep is needed for detection and an additional sweep is required for reconstruction. The quality obtained using SVM-M2 is higher since only nonnoisy pixels are used in the median. Both methods obtain best results using the nonrecursive method; that is, these methods are applied in an iterative way as shown previously.

### 4.2. Regression Based Filters

The regression method was presented previously in Section 3. The regression-based filters use SVM regression to obtain the reconstruction values. Although regression alone can be used as a reconstruction method, some blurring is present when only regression is used. To reduce the blurring effect, a previous noise detection stage is required and the SVM are used to perform this detection, as explained in Section 2.

The regression method is called SVM-R and only the recursive implementation method is used since the nonrecursive one gave poor results and the process was extremely slow.

## 5. Experiments and Results

Having defined the noise reconstruction filters, we will now present some results on reconstruction quality and conduct a comparison with other previously proposed methods. First, we define the measures of quality to be used and then we will present the reconstruction results obtained.

### 5.1. Quality Measures

It is pertinent at this point to define the quality measures used to adjust SVM detection and regression and to compare our proposed filters with other state-of-the-art filters.

*Reconstruction Quality*. In the field of image reconstruction several quality measures have been identified to determine whether the filter used is more or less efficient. Some of these measures include the following.

- *Mean Squared Error (MSE).* Better reconstruction gives lower values and zero indicates perfect reconstruction.
- *Peak Signal to Noise Ratio (PSNR).* This is a measure related to the previous one. It is a logarithmic measure widely used in image processing. Higher values indicate better quality.
- *Structural SIMilarity (SSIM).* This is a measure of quality that not only measures the difference between two images but also measures a number of structural parameters to check the similarity. These structural parameters are measured independently of local differences or the illumination changes of the image. A detailed description can be found in [34]. In this measure, a value near 1 indicates good quality, falling towards 0 as the quality decreases. The main advantage of this quality index is that it does not give so much importance to specific reconstruction errors but does give importance to the structural quality as a whole, besides having a direct relation to the subjective assessment of quality.

Finally only *PSNR* and *MSSIM* were used since *PSNR* is useful to compare the results with other studies which used *PSNR* or *MSE* and *MSSIM* gives a near subjective measure of quality.

*Noise Detection Quality.* To assess detection quality, we propose measuring the accuracy defined in the next equation:

(1) $$\begin{array}{c} \text{Accuracy}=100\cdot \frac{t_{p}+t_{n}}{t_{p}+f_{p}+f_{n}+t_{n}}, \end{array}$$

where *t*_*p*_ is the number of true positives, *t*_*n*_ is the number of true negatives, *f*_*p*_ is the number of false positives, and *f*_*n*_ is the number of false negatives.

This measure is given in percentage and, ideally, should be 100%. This ideal case is obtained when nor false positives neither false negatives are detected.

*Noise Regression Quality.* When regression is applied to every pixel in the image, the result is an approximation that can be directly compared with the original image. The better the reconstruction, the lower the error between the original and the approximated image. This error is measured using the *MSE* function described earlier.

### 5.2. Images Used in the Tests

We have used 10 well-known test images used in several noise reduction studies [5, 22, 35]. Four of them have been used in the classification and regression tests as well as in the quality results. Lenna is widely used to compare denoising algorithms and presents no major problems. It serves to conduct a comparison with the many other algorithms that use it. Albert is challenging due to the texture of the suit, but mostly because of the white collar where detection of noise becomes more difficult. Bridge presents some problems in two areas, one near the white bridge and the bridge itself and one black area which is the result of a bad scan. Barbara is quite challenging due to the large number of repeated textures and patterns it contains. Reconstruction of the lines is very difficult for all noise reduction algorithms.

The set is completed with 6 other known images like Airfield, Baboon, Boats, Goldhill, Lake, and Peppers.

### 5.3. Tuning SVM Classification Parameters

The first training step consists in defining the SVM kernel and its related parameters [28, 36]. Several kernels were tested, but only Gaussian kernels yielded competitive performances and a reduced set of support vectors. Thus, the parameters to be adjusted were *γ* and the regularization parameter (*C*).

In the noise detection training, *C* was established regarding the final number of support vectors since it is clear that the effect on classification is less relevant than this from *γ*. The value selected was *C* = 1000, since lower values increased the number of support vectors and higher values did not represent any improvement. *γ* appears in Gaussian kernel expression:

(2) $$\begin{array}{c} k\left(x,y\right)=\exp\left(-\gamma {||x-y||}^{2}\right). \end{array}$$

**Figure 5:**
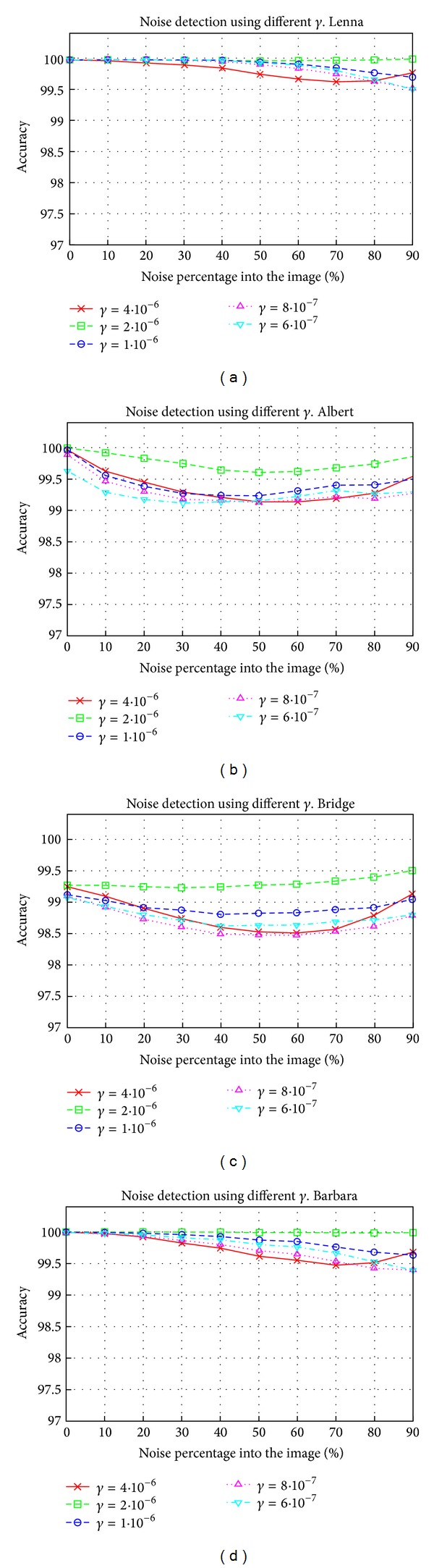
Noise detection results for different values of *γ*. The parameter presented is the accuracy that is related to the percentage of noise in the image. The results are shown for the Lenna, Albert, Bridge, and Barbara images.

Several classification tests were performed to adjust the value of *γ*. In these tests we used training images with *H* = 32 and *N* = 40%. In Figure 5 we show the results obtained for different values of *γ*. The best results were obtained for *γ* = 2 · 10^−6^ since for the entire range of noise the accuracy is not less than 99.5% for Lenna, 99.1% for Albert, 98.5% for Bridge, and 99.4% for Barbara. Obviously, different images gave different results and, for example, in our case the results for Albert or Bridge were worse than those for Lenna or Barbara. But we would like to highlight the fact that the worst result reached an accuracy of 98.5%. It must be noticed that the result obtained is independent from the images used to show the results.

### 5.4. Comparison of SVM Detection in Different Images

**Figure 6:**
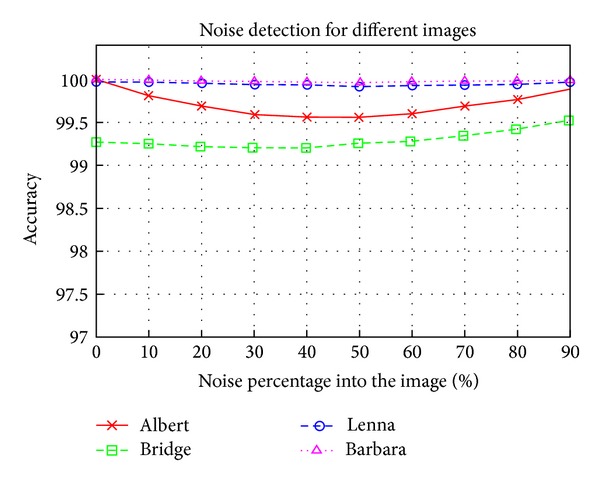
Detection results for the images used in our test.

At this point, we can show how the proposed impulse noise detection performs with noisy images.  Figure 6 shows the results for the images used in our tests. It is clear that the results for Lenna and Barbara were better than those for Albert and Bridge. It is noticeable that for Bridge even without noise a certain percentage of noisy pixels were detected, although for the other images, where no noise existed, no pixels were detected as noisy (Albert and Barbara) or only very reduced set was detected, as in the Lenna image.

An explanation of what is happening in the detection is given in the previously presented Figure 2.  Figure 2(a) shows the Lenna image with a 20% added noise and Figure 2(b) shows (marked as white pixels) the pixels detected as noisy. It is clear that the detection proposed performs well and there is no obvious correlation between image information and the noise detected.  Figure 2(c) shows which detected pixels are not actually noisy (false positives), which in this case only represented 0.04% of the entire image. The noise added to the Albert image (Figure 2(d)) was detected as well (Figure 2(e)), but there are some areas in the image that present a clear correlation with the original image, namely, some parts of the white collar. This effect is clearly evident in Figure 2(f), which shows falsely detected pixels. Nevertheless, these falsely detected pixels only represented 0.26% of the whole entire image. Thus Figure 2 shows that most of the noisy pixels were correctly detected and that the method proposed ensures a good detection ratio. For Bridge (Figure 2(i)), the results were worse due in part to central white areas but mainly to a black area near the bottom edge. Even so, the falsely detected pixels only represented the 0.76% of the image pixels.

Thus Figure 2 shows that most of the noisy pixels were correctly detected and that the method proposed ensures a good detection ratio.

### 5.5. Effect of Training Image Noise and Size on Detection

When we defined the training images, we stated that they can be varied in size and percentage of noise added. These two parameters are critical for detection quality and execution time. The greater the size, the larger the number of gray values and the higher the number of noisy pixels and hence the greater the number of example vectors required for training. Larger images are closer to real examples but entail an increased number of support vectors. The percentage of noise added to training images implies that a greater number of noisy pixels appear and, therefore, training will be more complete. However, the amount of noise should not be too great because, otherwise, noisy pixels can appear grouped together without any gray pixel nearby, which worsens rather than improves the results.

**Figure 7:**
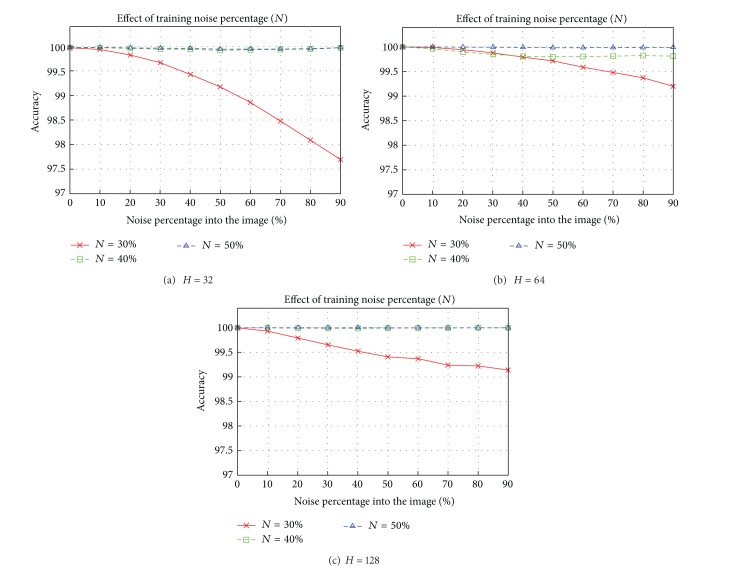
Noise detection results for different noise ratios using the Lenna image. The results are presented for different training image sizes.

Figure 7 shows the classification results for the Lenna image using different training image sizes and different percentages of noise in training images. The SVM parameters used were the best of those obtained in Section 5.3. It is clear that the best results for *N* were obtained 40% and 50% and that the best size was *H* = 128 pixels (although the difference with the other heights was not significant). With these results, the choice should be *H* = 128 and *N* = 50%. But this choice gives a high number of support vectors, as shown in Table 2.

### 5.6. Detection Complexity

One of the known disadvantages of SVM is the computational cost specially using a kernel like in this case. The number of operations needed to apply the trained classifier can be extracted by analyzing the classification function obtained:

(3) $$\begin{array}{c} f\left(x\right)=\overset{N_{\text{SV}}}{\underset{i}{\sum }}\alpha_{i}y_{i}\exp\left(-\gamma {||x_{i}-x||}^{2}\right). \end{array}$$

If we call *N*_*F*_ the number of features of the vector to classify and *N*_SV_ the number of support vectors obtained, for the evaluation of this classification function we need, for every support vector:

- *N*_*F*_ differences,
- *N*_*F*_ products,
- *N*_*F*_ − 1 additions,
- 2 products and an exponential that can be irrelevant compared to previous operations.

Then the number of operations needed is

(4) $$\begin{array}{c} \#\text{Ope}\approx N_{\text{SV}}\times 3\times N_{F}. \end{array}$$

This is the reason why sometimes [37] it is said that the run-time complexity of kernel methods using an RBF kernel is *O*(*N*_SV_ × *N*_*F*_). Remember that in our case *N*_*F*_ is the window size used in our algorithm and thus this size has been elected 3 × 3. It is clear that the greater the number of support vectors, the higher the execution time.

Obviously, the overall execution time is influenced by the image size (the classification function is applied over every pixel) and by the actual noise present. More noise implies more reconstruction operations that, in the case of the median, has a worst case complexity of *O*(*N*_*F*_) and in the case of SVM regression has, once again, a complexity of *O*(*N*_SV_ × *N*_*F*_).

Thus, our training choice must take into account a tradeoff between detection quality and speed. Looking for a reduced number of support vectors while obtaining good reconstruction results, more different training examples can improve detection but at the expense of a higher execution time.

There are a number of well-established techniques to reduce dimensionality (PCA, LDA, etc.) [38] that could be used in this case but at the expense of some lost information. To reduce the number of support vectors is important the correct tunning of C and gamma parameters, not only for good classification results but looking the smallest number of support vectors possible.

### 5.7. Tuning SVM Regression Parameters

As with classification, in SVM regression the training parameters must be tuned [39], including the kernel parameters. We summarize our choices below.

**Figure 8:**
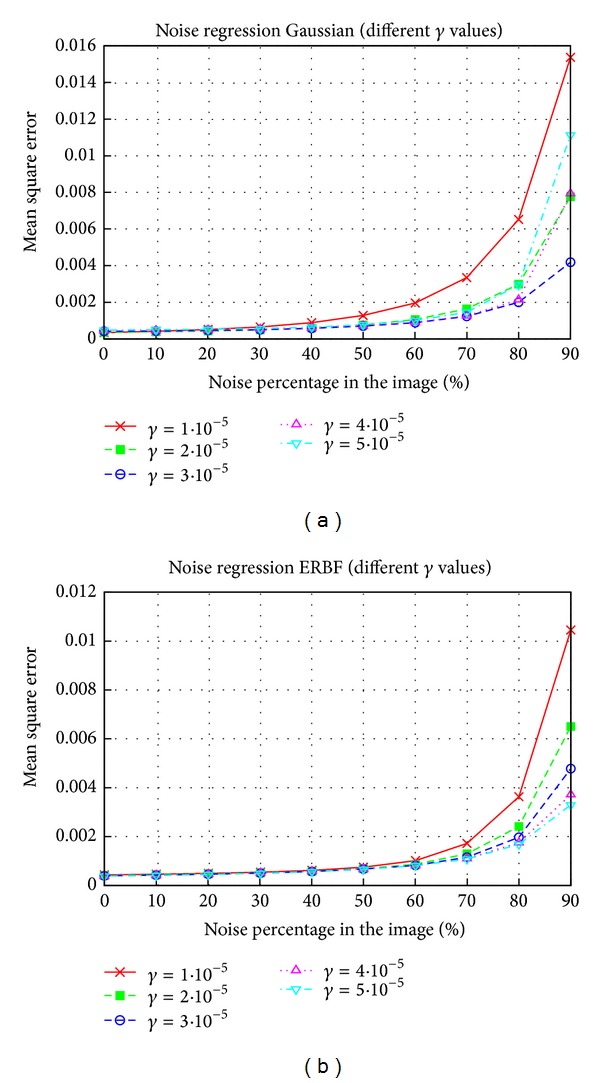
Mean square error for different values of *γ* in Gaussian and ERBF kernels.

1. *Kernel*. Several kernels were explored, including Gaussian, ERBF (exponential radial basis function), *χ* ^2^, and splines [39]. However, the results indicated that only Gaussian and ERBF would be useful given the reconstruction results and the number of support vectors obtained.
2. *Regularization Parameter C*. After an analysis with the selected kernels, it was clear that, by increasing *C*, the quality is increased until *C* = 100 and then it is reduced. The number of support vectors required is affected by *C* and is reduced as *C* is increased. Thus, we chose *C* = 1000 because this value yields a similar quality to *C* = 100 but implies a reduction of nearly 10% in the amount of support vectors necessary.
3. *Insensibility Parameter ε*. There is no noticeable difference between the reconstruction results for different values of *ε*. The only effect is on training speed and to increase this speed, the value chosen was *ε* = 10^−3^.
4. *Kernel Parameter γ*. In Figure 8, reconstruction results are shown for different values of *γ* in Gaussian and RBF kernels. The best value for the Gaussian kernel was *γ* = 3 · 10^−5^ and for the ERBF it was *γ* = 5 · 10^−5^. The results for the ERBF kernel are slightly better than for the Gaussian kernel, the only difference being that the number of support vectors is greater with the ERBF kernel.

### 5.8. Effect of Training Image Noise and Size on Regression

Size and percentage of noise are important parameters for the training images. Size increments increase the number of training vectors and the range of gray values, so better results are to be expected. However, the number of support vectors will also be increased and thus speed will be reduced. The effect of the noise added to training images is not clear, but similar noise ratios to those in corrupted images can be initially chosen.

**Figure 9:**
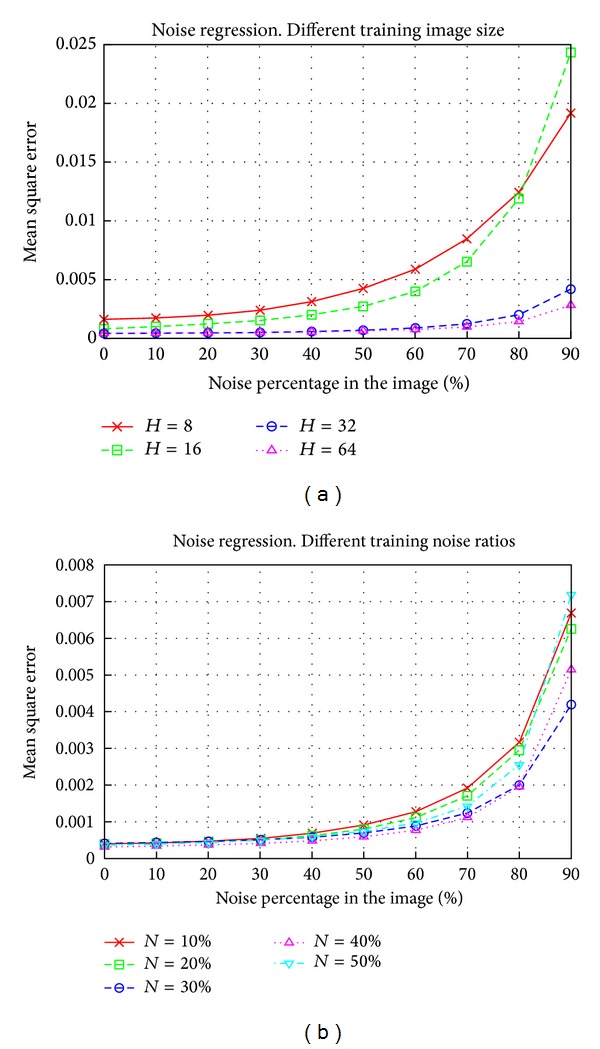
Regression results for different sizes and percentages of noise in training images. Size results were obtained with *N* = 30% and noise results were obtained using *H* = 32. The image used was Lenna and the SVM used the Gaussian kernel.

To obtain the best sizes and noise ratios for training images, several results for regression were obtained. In these tests, either size or noise was fixed and the other parameter was swept.  Figure 9 shows the results obtained for the Lenna image. To obtain noise results, *H* was set at 32, whilst for size results, *N* was fixed at 30%, since these were the best choices in each case. The results for size showed that size increments increased quality, but that this increase in quality occurred at the expense of increasing the number of support vectors. For example, with *H* = 32 we obtained 1192 support vectors, whereas using *H* = 64 these vectors increased to 3184. However, the quality did not increase by the same amount. The figures for noise showed that a ratio between 30% and 40% gave similar results. The support vectors obtained for these ratios were 1192 for 30% and 1396 for 40%; thus both of these ratios would be useful and there is no clear choice. The complexity issues discussed previously for classification apply for regression too.

### 5.9. Comparison between State-of-the-Art Methods and the Proposed Filters

The methods chosen to conduct the comparison were the following.

- DBAIN: a decision based filter for highly corrupted images [20].
- MMEM: dpecifically designed for “salt and pepper” noise and high noise ratios [14].
- Modified median filters: ACWM [19], AMF [12], PSMF [17], SDROM [5], and MDBUTMF [24].
- Fuzzy filters: filters based on fuzzy classification and reconstruction like DSFIRE [21], NAFSM [23], or FIDRM [22].

These methods, together with those proposed in this paper, have been used to reconstruct noisy images at different noise ratios, and reconstruction quality was measured using *PSNR* and *MSSIM*. The results are shown in Tables 3 and 4. These results have been obtained using 10 different test images and varying the noise ratios from 10 to 90%. We present the mean value for each method in the whole image set at each noise ratio and, finally, the mean value in all ratios and images.

From an analysis of these data, we can draw the following conclusions.

1. None of the methods proposed in the literature presents a better performance in all scenarios, that is, for different added noise ratios or for all the images evaluated.
2. There is a correlation between *PSNR* and *MSSIM* as regards the quality of reconstruction (poor *PSNR* implies a poor *MSSIM*, but the best *PSNR* is not the best *MSSIM*), although there are slight variations. Nevertheless, both provide information for verification of quality.
3. The best method when the noise is high (or very high) is the MMEM and for low and medium is MDBUTMF.
4. The results obtained for the filters proposed in this paper were superior to those reported in the literature for a wide range of noise ratios. Specifically, for low and medium ratios, they outperform most methods compared. The results for high noise ratios were superior to those obtained for MDBUTMF and were close to those obtained using MMEM, which is specifically designed for high noise ratios.
5. The best total mean values were obtained by some of the proposed methods showing that their performance is maintained along the whole ratios.

**Figure 10:**
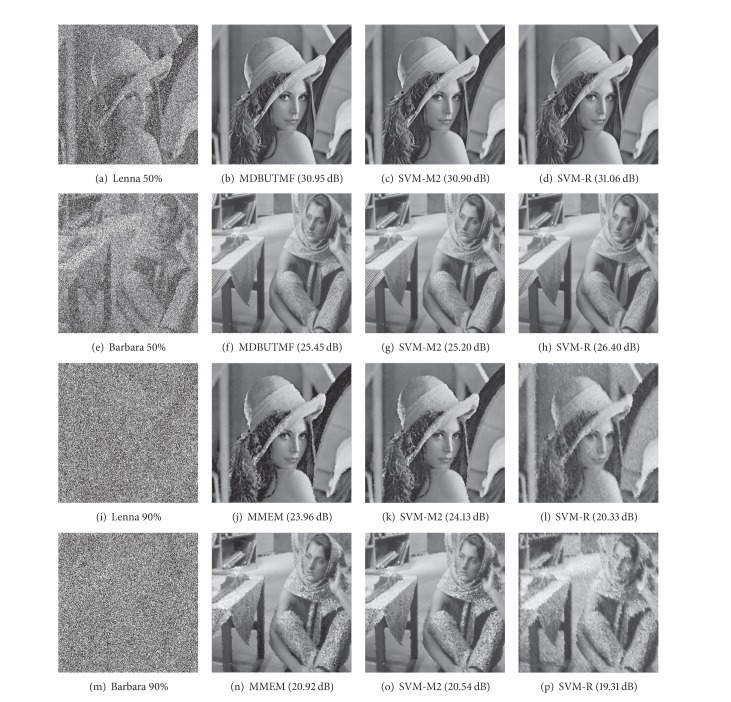
Reconstruction results for the Lenna and Barbara images with 50% and 90% of “salt and pepper” noise, using the MDBUTMF (50%), MMEM (90%), SVM-M2, and SVM-R methods.

Some reconstruction examples are shown in Figure 10 where MDBUTMF and MMEM are compared to SVM-M2 and SVM-R (more results for different methods and different images can be found in http://agamenon.tsc.uah.es/Investigacion/gram/papers/Noise/). For medium noise ratio (50%) MDBUTMF yielded the best mean measure, a value shown in Figure 10 the results can vary depending on the image. For a high noise ratio (90%), the image was reconstructed by MMEM and SVM-M2 but the recursive nature of SVM-R yielded poor results. It is clear that our methods maintain quality in a wide range of noise ratios.

### 5.10. Robustness of the Algorithm

One important issue that must be addressed when some kind of training is used is the robustness of the algorithm proposed since this training could give divergent results with slight changes.

To prove robustness of our proposed SVM algorithms we present some data in Table 5 where we show, for two different images (Lenna and Albert), the mean and the standard deviation measuring MSSIM and PSNR for 10 different noise patterns. Obviously, changes in noise patterns produce different results. But these results have a reduced standard deviation showing the robustness of our proposed algorithms.

## 6. Conclusions

In this paper we have presented a series of impulsive noise reduction methods based on SVM that can be applied to gray-scale images. We have studied their ability to detect noisy pixels using synthetic images for training and the possibility of reconstruction using regression based on SVM training and, again, synthetic images.

After checking the detection and reconstruction ability of the SVM, several filters have been proposed for detection or reconstruction or both. The proposed methods were compared both against each other and with state-of-the-art methods.

It has been shown that the proposed methods are a valid alternative and may even outperform other methods in the literature for a larger range of noise levels.

A great advantage of our proposed filters is that since the training samples are generated automatically, the training is customizable and furthermore it is easy and fast. The training based methods allow flexibility since adapting the training to the context can improve the results.

The use of SVM and the underlying well-funded probability relation between classification function and probability estimation [40] gives us a tool to apply this noise reduction method to other noise types. If the noise added is random valued or Gaussian, the probability estimation can give the degree of noise contamination instead of a classification label. This degree obtained can be used in the reconstruction process as in some fuzzy noise reduction methods.

On the other hand, the proposed scheme has a computational complexity higher than others compared in this paper. As an example, MMEM is really simple and a training is not needed to obtain good results for high noise ratios. The need of complex arithmetic operations in SVM implementation makes our scheme slower than other Decision Based Algorithms with less requirements.

However, we think that the proposed scheme is opened to be improved in several ways including training, implementation, and application to other noise types and currently can be successfully applied to the salt and pepper noise problem.

## Figures and Tables

**Table 1:** Example of regression training vectors (LIBSVM format) taken from images in Figure 3.

4	1 : 255	2 : 255	3 : 8	4 : 255	5 : 255	6 : 0	7 : 4	8 : 8
8	1 : 255	2 : 8	3 : 12	4 : 255	5 : 12	6 : 4	7 : 8	8 : 12
12	1 : 8	2 : 12	3 : 16	4 : 255	5 : 16	6 : 8	7 : 12	8 : 16
16	1 : 12	2 : 16	3 : 20	4 : 12	5 : 20	6 : 12	7 : 16	8 : 255
20	1 : 16	2 : 20	3 : 255	4 : 16	5 : 255	6 : 16	7 : 255	8 : 24

**Table 2:** Number of support vectors for different training image noise ratios and sizes. These numbers include support vectors for detecting both black and white noisy pixels.

*H*	*N*		
	30%	40%	50%
32	57	101	118
64	162	204	226
128	487	543	515

**Table 3:** Table results in mean PSNR for different impulse noise reduction methods. The mean is obtained for different noise ratios in 10 test images. Total column reflects the total mean for all ratios and images.

Method	Noise percentage									
	10	20	30	40	50	60	70	80	90	Total
ACWM [19]	31.24	27.49	22.82	18.48	14.89	12.10	9.78	7.94	6.47	16.80
AMF [12]	32.17	30.45	28.77	27.18	25.59	23.97	21.87	18.03	12.03	24.45
DBAIN [20]	36.58	32.97	30.51	28.50	26.61	24.80	22.90	20.85	18.05	26.86
DSFIRE [21]	34.14	30.13	25.69	21.10	17.14	13.93	11.30	9.23	7.60	18.92
FIDRM [22]	36.28	32.53	30.12	28.29	26.76	25.39	23.89	22.08	19.68	27.23
MMEM [14]	27.77	30.46	29.52	28.71	27.84	*26.88 *	*25.76 *	*24.45 *	*22.59 *	27.11
NAFSM [23]	34.26	31.31	29.54	28.17	27.05	26.03	25.01	23.81	21.22	27.38
PSMF [17]	26.35	25.84	25.08	23.97	21.95	18.91	14.98	9.67	7.61	19.37
SDROM [5]	30.92	28.93	27.07	25.19	22.97	20.53	17.62	14.34	10.79	22.04
MDBUTMF [24]	*37.22 *	*33.86 *	*31.62 *	*29.82 *	*27.94 *	25.53	22.45	18.83	15.05	26.92
SVM-M1 (32-40)	35.49	31.96	29.32	26.53	24.69	22.33	19.76	13.07	5.96	23.23
SVM-M1 (64-40)	35.57	32.15	29.49	26.87	25.10	22.80	20.12	13.28	5.97	23.48
SVM-M2 (32-40)	36.02	32.96	30.86	29.19	27.74	26.42	25.14	23.80	22.09	*28.25 *
SVM-M2 (64-40)	35.96	32.90	30.81	29.14	27.69	26.37	25.11	23.77	22.06	28.20
SVM-R (32-40)	35.63	32.71	30.76	29.14	27.70	26.25	24.71	22.71	19.63	27.69
SVM-R (64-40)	35.47	32.59	30.68	29.10	27.67	26.23	24.70	22.71	19.63	27.64

**Table 4:** Table results in mean MSSIM for different impulse noise reduction methods. The mean is obtained for different noise ratios in 10 test images. Total column reflects the total mean for all ratios and images.

Method	Noise percentage									
	10	20	30	40	50	60	70	80	90	Total
ACWM [19]	0.930	0.876	0.734	0.502	0.277	0.141	0.069	0.033	0.014	0.397
AMF [12]	0.922	0.909	0.884	0.847	0.800	0.742	0.653	0.466	0.139	0.707
DBAIN [20]	0.978	0.951	0.918	0.877	0.825	0.761	0.677	0.570	0.427	0.776
DSFIRE [21]	0.961	0.905	0.783	0.575	0.348	0.183	0.088	0.040	0.016	0.433
FIDRM [22]	0.977	0.948	0.912	0.870	0.821	0.767	0.696	0.597	0.456	0.783
MMEM [14]	0.943	0.918	0.893	0.868	0.837	0.801	*0.754 *	*0.693 *	*0.596 *	0.811
NAFSM [23]	0.968	0.938	0.904	0.868	0.828	0.784	0.732	0.665	0.538	0.803
PSMF [17]	0.856	0.836	0.802	0.754	0.671	0.509	0.272	0.045	0.019	0.529
SDROM [5]	0.925	0.883	0.829	0.754	0.645	0.508	0.343	0.179	0.067	0.570
MDBUTMF [24]	*0.979 *	*0.956 *	*0.930 *	*0.897 *	0.851	0.765	0.608	0.384	0.178	0.728
SVM-M1 (32-40)	0.978	0.950	0.912	0.859	0.802	0.723	0.617	0.366	0.077	0.698
SVM-M1 (64-40)	0.978	0.950	0.912	0.860	0.803	0.725	0.619	0.369	0.077	0.699
SVM-M2 (32-40)	0.978	0.954	0.926	0.893	0.853	*0.807 *	0.752	0.685	0.585	*0.826 *
SVM-M2 (64-40)	0.978	0.954	0.925	0.892	0.852	0.806	0.751	0.684	0.584	0.825
SVM-R (32-40)	0.976	0.953	0.926	0.894	*0.854 *	0.803	0.735	0.636	0.478	0.806
SVM-R (64-40)	0.976	0.953	0.926	0.894	*0.854 *	0.803	0.735	0.636	0.479	0.806

**(a):** 

PSNR results: Albert						
Noise %	SVM-M1		SVM-M2		SVM-R	
	μ	σ	μ	σ	μ	σ
10	37.806326	0.081000	37.634073	0.095187	37.806326	0.081000
20	34.012864	0.077311	34.247895	0.100104	34.012864	0.077311
30	31.334875	0.075222	32.111316	0.096603	31.334875	0.075222
40	28.782425	0.080101	30.524735	0.041627	28.782425	0.080101
50	27.047406	0.054255	29.098885	0.054799	27.047406	0.054255
60	24.689836	0.179538	27.809312	0.058029	24.689836	0.179538
70	22.478286	0.124136	26.584962	0.067960	22.478286	0.124136
80	15.891552	0.149735	25.358707	0.047150	15.891552	0.149735
90	8.109299	0.022287	23.885132	0.045141	8.109299	0.022287

**(b):** 

PSNR results: Lenna						
Noise %	SVM-M1		SVM-M2		SVM-R	
	μ	σ	μ	σ	μ	σ
10	40.210190	0.220927	40.647102	0.137564	40.483306	0.123692
20	35.529780	0.412996	36.987532	0.152243	37.014919	0.148138
30	32.294158	0.230317	34.537564	0.092911	34.751509	0.085640
40	28.576325	0.381850	32.600946	0.062708	32.823432	0.055126
50	26.726578	0.183652	30.849589	0.072874	31.000031	0.114136
60	23.778253	0.182517	29.311999	0.071502	29.154773	0.102010
70	20.483630	0.121690	27.827742	0.055517	27.053785	0.074279
80	12.017827	0.171127	26.298444	0.065581	24.520623	0.047364
90	4.453599	0.014529	24.109759	0.068257	20.390460	0.101721

**(c):** 

MSSIM results: Albert						
Noise %	SVM-M1		SVM-M2		SVM-R	
	μ	σ	μ	σ	μ	σ
10	0.970160	0.000260	0.970334	0.000257	0.969802	0.000227
20	0.932638	0.000469	0.936817	0.000381	0.936707	0.000548
30	0.884381	0.000755	0.899063	0.000610	0.900619	0.000888
40	0.821351	0.000890	0.855522	0.000718	0.860015	0.000879
50	0.756570	0.001241	0.804625	0.000673	0.813316	0.000822
60	0.672137	0.002908	0.746005	0.000909	0.758855	0.001158
70	0.579394	0.003105	0.679557	0.000984	0.691942	0.001030
80	0.368391	0.006010	0.604714	0.001191	0.604779	0.001498
90	0.093756	0.001267	0.510287	0.001127	0.476452	0.002035

**(d):** 

MSSIM results: Lenna						
Noise %	SVM-M1		SVM-M2		SVM-R	
	μ	σ	μ	σ	μ	σ
10	0.985231	0.000228	0.985689	0.000188	0.985842	0.000181
20	0.964831	0.000407	0.969028	0.000366	0.969652	0.000358
30	0.936021	0.000549	0.949325	0.000391	0.950161	0.000462
40	0.891417	0.003147	0.925923	0.000477	0.926024	0.000398
50	0.847043	0.001220	0.896965	0.000344	0.893932	0.000713
60	0.777944	0.002245	0.862579	0.000881	0.850796	0.001099
70	0.679344	0.002105	0.820914	0.000564	0.788319	0.001127
80	0.386960	0.004851	0.768412	0.000831	0.693430	0.001324
90	0.046905	0.000822	0.687110	0.000864	0.526401	0.002583
